# Microfluidic Single-Cell Proteomics Assay Chip: Lung Cancer Cell Line Case Study

**DOI:** 10.3390/mi12101147

**Published:** 2021-09-23

**Authors:** Yugyung Jung, Minkook Son, Yu Ri Nam, Jongchan Choi, James R. Heath, Sung Yang

**Affiliations:** 1 Department of Biomedical Science and Engineering, Gwangju Institute of Science and Technology (GIST), Gwangju 61005, Korea; jyg4789@gist.ac.kr (Y.J.); minkook87@naver.com (M.S.); yurinam@kaist.ac.kr (Y.R.N.); 2 Department of Chemistry, Korea Advanced Institute of Science and Technology (KAIST), Daejeon 34141, Korea; 3 School of Mechanical Engineering, Gwangju Institute of Science and Technology (GIST), Gwangju 61005, Korea; jongchan.choi@isbscience.org; 4 Institute for Systems Biology, Seattle, WA 98109, USA; jim.heath@isbscience.org; 5 Department of Bioengineering, University of Washington, Seattle, WA 98105, USA

**Keywords:** microfluidics, cancer, proteomics, heterogeneity, lung cancer

## Abstract

Cancer is a dynamic disease involving constant changes. With these changes, cancer cells become heterogeneous, resulting in varying sensitivity to chemotherapy. The heterogeneity of cancer cells plays a key role in chemotherapy resistance and cancer recurrence. Therefore, for effective treatment, cancer cells need to be analyzed at the single-cell level by monitoring various proteins and investigating their heterogeneity. We propose a microfluidic chip for a single-cell proteomics assay that is capable of analyzing complex cellular signaling systems to reveal the heterogeneity of cancer cells. The single-cell assay chip comprises (i) microchambers (*n* = 1376) for manipulating single cancer cells, (ii) micropumps for rapid single-cell lysis, and (iii) barcode immunosensors for detecting nine different secretory and intracellular proteins to reveal the correlation among cancer-related proteins. Using this chip, the single-cell proteomics of a lung cancer cell line, which may be easily masked in bulk analysis, were evaluated. By comparing changes in the level of protein secretion and heterogeneity in response to combinations of four anti-cancer drugs, this study suggests a new method for selecting the best combination of anti-cancer drugs. Subsequent preclinical and clinical trials should enable this platform to become applicable for patient-customized therapies.

## 1. Introduction

Cancer is a dynamic disease that becomes increasingly heterogeneous as it progresses [1,2,3]. Cancer cells remain unstable, even after becoming malignant, and constantly change by acquiring a variety of mutations [2]. With these continuous changes, cancer cells produce bulk tumors formed from groups of heterogeneous cells, which have various sensitivities to chemotherapy [3]. The heterogeneity of cancer cells contributes to the small number of drug-tolerant cancer cells. Those that survive chemotherapy eventually play a key role in chemotherapy resistance and cancer recurrence [4,5,6]. Therefore, heterogeneity in tumors has recently emerged as a critical factor in the diagnosis and treatment of cancer [7]. Furthermore, it was shown that intra-tumor heterogeneity can be used as one of the clinically important prognostic factors [8,9,10]. This implies that patients with tumors in which the heterogeneity is high are more likely to have a poor prognosis for chemotherapy and a higher resistance to targeted treatment [11]. In the clinical setting, the most commonly used method for chemotherapy-resistant tumor cells is a combination of two or more anti-cancer drugs, potentially lowering the incidence of various drug-tolerant cancer cell groups due to the heterogeneity of the tumor [6]; for such strategies to be successful for cancer patients, it is important to find the most effective combination and appropriate dose for anti-cancer drugs. Therefore, it is necessary to accurately diagnose the heterogeneity of cancer cells and predict the effectiveness of particular chemotherapies or combination therapies before applying customized therapy to each patient [12,13].

Lung cancer, which has the highest mortality rate among all tumors, has become an important target for personalized medicine [14]. As genomic sequencing technology has developed, various genetic data have been used to select personalized drugs [15]. Although there have been several successes with this approach, cancer genomics is very complex, and genes alone cannot predict all protein modifications and expressions. This limits the understanding of drug responses [16,17]. Single-cell proteomic analysis can confirm protein heterogeneity by revealing correlations among proteins that cannot be predicted by genomic data analysis [18,19]. Therefore, to determine the appropriate treatment for each patient, it is necessary to analyze the genomic and proteomic data together [20]. Many single-cell proteomics tools for revealing the response to drugs have been studied. Nanostructure initiator mass spectrometry (NIMS) is a representative example [21]. NIMS enables the quantitative assessment of drug interactions and reactions via cell and tissue biomarker measurements [22]. However, this technology requires a large laboratory infrastructure and is not easily accessible. In addition, many proteomic studies have used microfluidics [23,24,25]. However, these methods are limited by their low throughput, small number of measurable proteins, and inability to simultaneously confirm the internal signal systems and secretion proteins [26,27,28,29]. Therefore, a highly efficient analysis method is required to simultaneously measure the multiple signaling molecules associated with the proliferation and necrosis of cancer cells treated with multiple combinations of anti-cancer drugs at the single-cell level.

Epidermal growth factor receptor (EGFR) mutations are the most commonly observed mutations in non-small cell lung cancer and EGFR tyrosine kinase inhibitors (gefitinib and erlotinib) are used as the primary treatment [30]. However, 66% of patients develop cancer recurrence with T790M secondary mutations [31,32]. In these cases, osimertinib is proven to be effective against T790M mutations [32,33]. In addition to osimertinib, studies have been conducted on the phosphatidylinositol 3-kinase (PI3K)/protein kinase B (AKT) pathway, the Janus kinase (JAK) pathway, and the mitogen-activated protein kinase (MAPK) pathway, which correspond to the EGFR downstream pathways [34,35,36].

In this study, we propose a single-cell proteomics assay, comprising a microfluidic platform that can analyze cancer signal transduction systems to elucidate the heterogeneity of cancer cells. Using a single-cell assay chip, we analyzed and evaluated the effects of combinations of four targeted anti-cancer drugs: Osimertinib (O, PI3K inhibitor), LY294002 (L, PI3K inhibitor), Ruxolitinib (R, JAK inhibitor), and Selumetinib (S, MEK inhibitor). These are applied to non-small cell lung cancer cell lines with EGFR and T790M mutations that are resistant to primary drugs.

## 2. Materials and Methods

**Figure 1:**
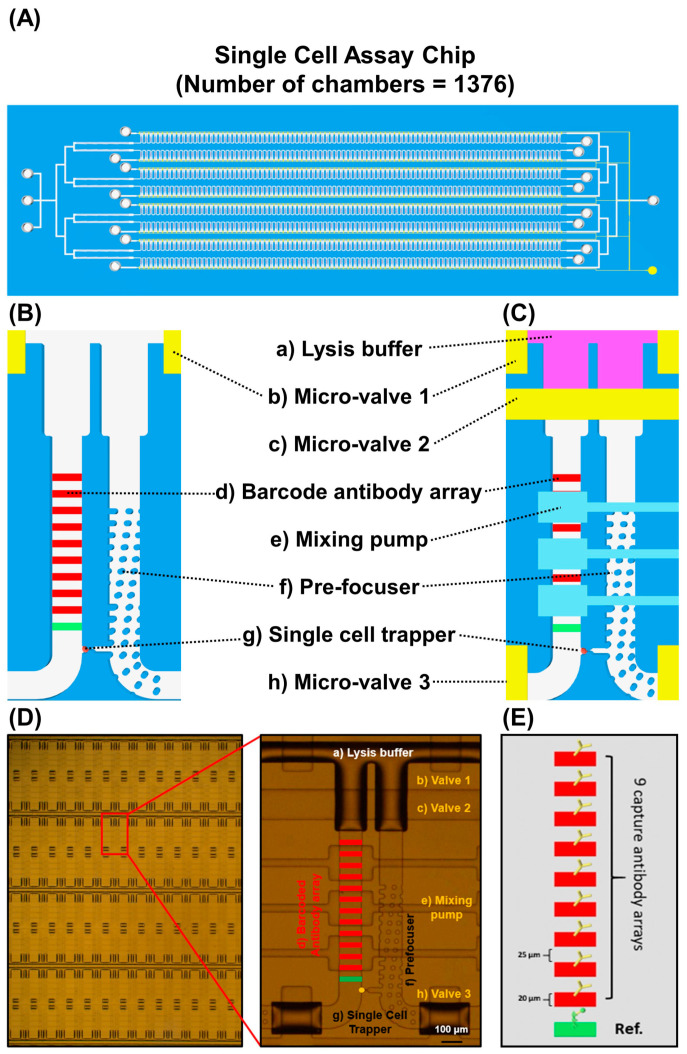
Design of single-cell proteomics assay chip. (**A**) Schematic drawing of the single-cell assay chip. (**B**,**C**) A schematic view of a single chamber. (**D**) Real image of single-cell proteomics assay chip. (**E**) Schematic view of multiplexed capture antibody arrays comprising a reference probe (green) and captured antibody arrays (red).

The single-cell assay chip comprises (i) microchambers (*n* = 1376) for manipulating single cancer cells, (ii) micropumps for rapid single-cell lysis, and (iii) barcode immunosensors for detecting nine different secretive and intracellular proteins. The device used in this study comprises two layers (flow and pump/valve layers) made of polydimethylsiloxane (PDMS) with a bottom substrate that has been integrated with multiple immunosensor arrays. The single-cell assay chip had 1376 microchambers for single-cancer-cell manipulation (Figure 1A). A schematic view of a single chamber is shown in Figure 1B,C. A microscopic image of the chamber is shown in Figure 1D. The pneumatic microvalves and mixing pumps allow for rapid single-cancer-cell lysis and washing. The barcode immunosensor array, comprising one reference and nine different proteins (p-AKT, p-P70S6K, p-ERK, p-STAT3, p-P53, CPS3, MMP2, VEGF, and M-CSF1) involved in cancer cell proliferation and necrosis, was integrated to allow all protein concentration levels to be simultaneously obtained (Figure 1E). The working principle of the microfluidic single-cell proteomics assay chip is shown in Supplementary Materials Video S1.

### 2.1. Device Preparation

Each layer in the single-cell microfluidic platform consisted of PDMS (Sylgard 184^®^, Dow Corning, Midland, MI, USA) mixed in a 10:1 ratio with a base and curing agent. For the control layer, PDMS was cast at a height of 3 mm. For the flow layer, PDMS was spin-coated at 2000 rpm on a dual-patterned photoresist mold. After curing the PDMS layer, each layer was cleaned with tape (Scotch^®^ Magic^TM^, St. Paul, MN, USA) before being treated with oxygen plasma at 100 W for 30 s in a plasma machine (Femtoscience, Hwaseong, Korea). The two-layer PDMS assembly was formed by bonding the layers using an oxygen plasma treatment. The device was finalized by assembling the multiplex barcode substrate via physical contact between the two sides.

### 2.2. Preparation of the Multiplex Barcode Array

The multiplex barcode array contains ten stripes, including one reference probe and nine capture antibody probes. All antibodies used in the multiplex barcode array were obtained from commercial enzyme-linked immunosorbent assay (ELISA) kits (Supplementary Materials Table S1; R&D Systems, DuoSet^®^, Minneapolis, MN, USA). Each contains capture antibodies, detection antibodies, and standard proteins. To immobilize the capture antibodies on a glass substrate, its surface properties must be modified. First, the glass substrate was treated with oxygen plasma (100 W, 20 sccm, 30 s). Then, by treating the substrate with 3-aminopropyltriethoxysilane (APTES) solution (3% *v/v*) in ethanol overnight, the surface was converted to an amine surface, which can be converted into an aldehyde surface using glutaraldehyde (10% *v*/*v*) solution. The amine groups of the capture antibody bind to aldehyde groups. The procedure is illustrated in Supplementary Materials Figure S1. The PDMS chip for the barcode array comprised ten microfluidic channels. The chip and aldehyde-treated surfaces were combined by physical contact. Each capture antibody was loaded into the inlet of the PDMS chip and then introduced into the array by a vacuum pump. After incubation for 12 h at 4 °C, each channel was washed with phosphate-buffered saline (PBS). After the PDMS chip was peeled off, the barcode antibody array was ready to be tested. Then, the final PDMS channel, including microchamber and micropumps, was bonded to the glass chip with antibody coating.

### 2.3. Cell Line and Reagents

For single-cell assay chip validation, a non-small-cell lung cancer cell line (NCI-H1650), which has a deletion mutation in EGFR exon 19, was obtained from the Korean Cell Line Bank (Seoul, Republic of Korea). For the proteomics experiments, a non-small-cell lung cancer cell line (NCI-H1975) was obtained from ATCC (USA). The cells were grown in Roswell Park Memorial Institute (RPMI) 1640 medium (Gibco, Grand Island, NE, USA). To produce a complete growth medium, 10% fetal bovine serum (Atlas, Fort Collins, CO, USA) and 1% penicillin streptomycin (Gibco, Grand Island, NE, USA) of the final concentration were added to the medium. The cells were incubated in a humidified incubator at 37 °C with 5% CO_2_.

### 2.4. MTT Assay and Drug IC50 Measurement

3-(4, 5-dimethylthiazol-2-yl)-2, 5-diphenyltetrazolium bromide (MTT) (Sigma-Aldrich, St. Louis, MO, USA) was used for the MTT assay. The used drugs (Selleckchem, Houston, TX, USA) were: Osimertinib (EGFR inhibitor), LY294002 (PI3K inhibitor), Selumetinib (MEK inhibitor), Ruxolitinib (JAK inhibitor). In this study, a general protocol for an MTT assay was used to evaluate the half-maximal inhibitory concentration (IC_50_) of each drug (O, L, R, S) and the drug combination for the H1975 lung cancer cell line (Supplementary Materials Figure S2). A particular drug combination was added to a 96-cell culture plate (100 μL well^–1^). The cancer cell suspension (H1975) was added at a density of 4000 cells per well (4 × 10^4^ mL^–1^). The cell culture plate was then incubated for 72 h at 37 °C with 5% CO_2_. After 72 h, 10 μL of MTT solution (5 mg mL^–1^) was added to each well and the plate was incubated at 37 °C with 5% CO_2_ for 2 h. After incubation, the culture medium was completely removed and 200 μL of dimethyl sulfoxide (DMSO) was added to each well to dissolve the formazan. The plate was placed on an orbital shaker for 10 min and the absorbance was measured at 570 nm. After testing, values of relative cell proliferation (%) with respect to the control group, for various drug concentrations, were calculated using Equation (1) [37].

(1) $$\mathrm{Relative}\;\mathrm{proliferation}\;(\%)=100-(100\times \frac{{\mathrm{OD}}_{\mathrm{control}}\;-{\mathrm{OD}}_{\mathrm{sample}}}{{\mathrm{OD}}_{\mathrm{control}}})$$

Drug combinations generated from osimertinib, LY294002, ruxolitinib, and selumetinib were tested using the MTT assay to determine the IC_50_ concentration of each inhibitor. For the dual-drug combinations, the IC_50_ concentration of each drug was set at 100% and diluted with culture medium (RPMI-1640) to produce 0%, 25%, 50%, and 75% relative proliferation values (Supplementary Materials Figure S3). For the triple-drug combinations, the mixtures comprised dual-combination drugs combined with another single drug. First, the low viability of the dual combination was ranked. Based on the results, three combinations of osimertinib + ruxolitinib + LY294002 (ORL), osimertinib + ruxolitinib + selumetinib (ORS), and osimertinib + LY294002 + selumetinib (OLS) were prepared. Relative cell proliferation was calculated for the given drug combination compared to the control group.

### 2.5. Single-Cell Assay

The protocol for the single-cell assay was as follows. First, lung cancer cells (5 × 10^5^ cells mL^–1^) were introduced into the single-cell assay chip using a syringe pump (Cetoni GmbH, Korbussen, Germany) at a flow rate of 50 μL h^–1^. Eight different drug combinations, with the culture medium as a control, were introduced (50 μL h^–1^) into each branch, with each providing treatment for 172 single-cell assay chambers. After the cells and drugs were introduced, each single-cell assay chamber was independently isolated using a pneumatic valve. The assay chip was incubated for 12 h for the drug treatment. After incubation, a cell lysis buffer was introduced and mixed using an automated procedure controlled by a Laboratory Virtual Instrument Engineering Workbench (LabVIEW 2017, National Instruments, Austin, TX, USA) program. Once the cell membrane ruptured, chamber washing was performed to avoid contamination from other single-cell chambers. A cocktail of detection antibodies (3 μg mL^–1^), a mixture of streptavidin-Cy5 (1 μg mL^–1^), and a single strand of cDNA-Cy3 (50 nM) were sequentially introduced. Signal amplification using biotinylated anti-streptavidin antibodies was used to increase the fluorescence signal (Supplementary Materials Figure S4). The finalized assay chip was scanned using a GenePix scanner (Molecular Devices, San Jose, CA, USA) at 100% (635 nm for Cy5) and 33% (532 nm for Cy3) laser power levels, with optical gains of 700 and 500, respectively, to extract the fluorescent intensity.

### 2.6. Single-Cell Data Analysis

All figures were created using Prism8 (GraphPad Software Inc., San Diego, CA, USA), Python version 3.7 (Python Software Foundation, Wilmington, DE, USA), and Anaconda (Anaconda, Inc., Austin, TX, USA). Principal component analysis (PCA), a technique for reducing the dimension of data, expanding the interpretability by minimizing information loss [38], was performed using Python version 3.7 and Anaconda, and cluster analysis was performed using R version 3.6.0 (R foundation for Statistical Computing, Vienna, Austria). To create the heatmap, all cancer cell data were collated and transformed using RobustScaler in Equation (2), which returns a median value that is less sensitive to outliers [39].

(2) $$\mathrm{RobustScaler}\;=\;\frac{X\;-\;\mathrm{median}}{\mathrm{interquartile}\;\mathrm{range}}$$

The median value for each protein among the cancer cell groups that had undergone anti-cancer drug treatment was standardized to the RobustScaler, using the median value and interquartile range (IQR) of the control group. For each drug, a heat map was created that corresponded to three experiments, as well as a heat map corresponding to the average of each drug group. The two best drug groups, with the greatest overall protein loss (OL100 S100, OR50 L50), and the two worst drug groups, having the lowest overall protein loss (O50 R100, O50 R50), were selected. Heat maps of single cancer cells were created for the selected groups. An independent Student’s *t*-test was used to compare protein secretion between the drug and control groups. The mean protein changes for each protein in the best and worst drug groups were calculated using the RobustScaler. Histograms for each protein, for one of the best (OL100 S100) and worst (O50 R100) drug groups, were also calculated using RobustScaler. The Pearson correlation coefficient was calculated for protein correlation analysis.

### 2.7. Heterogeneity Evaluation and Analysis

Standardized RobustScaler data were used for heterogeneity evaluation and PCA was used to visually depict the heterogeneity of cancer cells [40]. Hierarchical clustering analysis was used to quantitatively evaluate the heterogeneity [41]. The similarity between single cells was measured using Euclidian distance dissimilarity coefficients with Ward’s minimum variance method. The calculated dissimilarity coefficients were used to measure the heterogeneity of cell populations.

### 2.8. ELISA Assay

For the bulk ELISA test, cells were cultured on the cell culture plate and a sandwich ELISA test was conducted using a commercial ELISA kit (R&D Systems, DuoSet^®^, Minneapolis, MN, USA). The absorbance of each target was measured using ELISA reader. Nine different molecules (p-AKT, p-70S6K, p-ERK, p-STAT3, p-P53, cleaved caspase 3, MMP2, VEGF, and M-CSF1) were measured. For the test target, the supernatant was collected before the cell lysate and the lysis was then collected.

## 3. Results

### 3.1. Validation of Single-Cell Proteomics Assay Chip Using H1650 Lung-Cancer Cell Line

**Figure 2:**
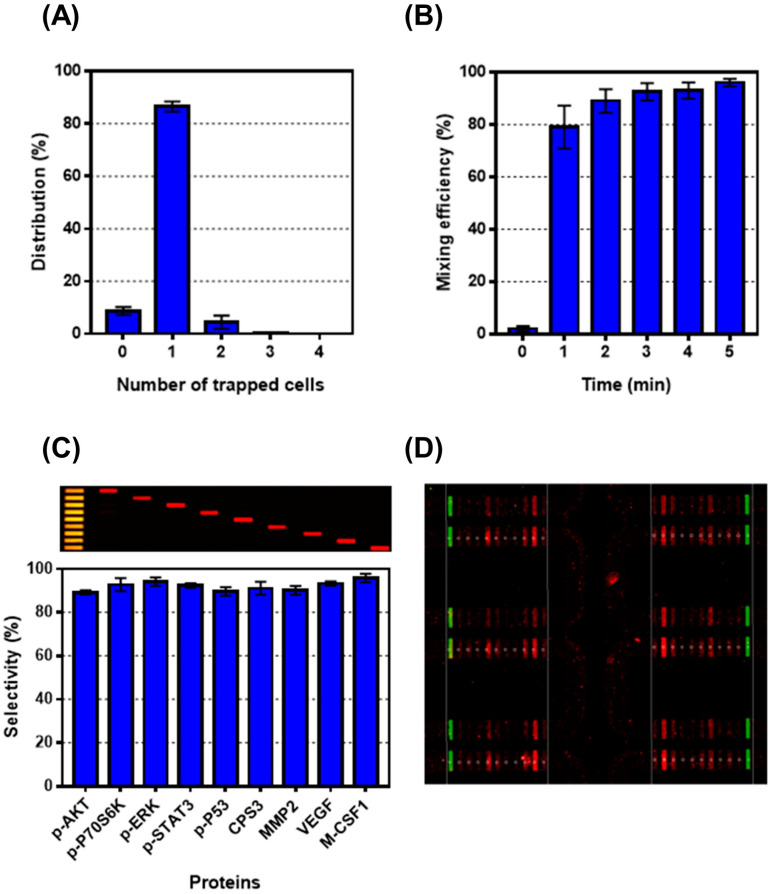
Validation of single-cell proteomics assay chip. (**A**) Of the 1376 chambers, 98.9% (±0.15%) acquired cancer cells and 89.5% (±3.7%) of the trapped cells were verified as single cells. (**B**) High (>95%, within 5 min) mixing efficiency was achieved. (**C**) Fluorescent image of a cross-reactivity result (upper) is shown, and selectivity values are shown with high selectivity (>89.5%) between the target proteins and corresponding antibody (lower). (**D**) Fluorescent image of multiplexed antibody array. The reference probes are green while the capture antibody arrays are red.

The single-cell assay chip was experimentally validated and optimized using the H1650 lung cancer cell line. First, each cancer cell was localized using a pre-focusing microstructure based on deterministic lateral displacement and a subsequent cell-trapping microstructure. Of the 1376 chambers, 98.9% (±0.15%) acquired cancer cells, either in the form of single cells or multiple cells (up to four cells), and 89.5% (±3.7%) of the trapped cells were verified as single cells (Figure 2A). Second, using the peristaltic pump, which allows for uniform washing and mixing of the proteins from the lysed cell, rapid single-cell lysis was demonstrated. As a result, a high (>95%, within 5 min) mixing efficiency was achieved, as shown in Figure 2B. Rapid single-cell lysis and individual chamber washing were achieved not only in a single chamber but also in the entire assay chambers, without any fluidic interference or contamination among the chambers (Supplementary Materials Figure S5). Finally, the selectivity of the multiplex assay was validated using ten different types of antibodies: one as a reference probe and nine probes for different proteins. As a result, the barcode immune sensor exhibited high selectivity for each protein (>89.5%) (Figure 2C). After trapping each lung cancer cell into an individual chamber, seven drug cocktails and one control were applied per device and the fluorescent signal intensities were measured using a GenePix scanner; the corresponding changes in the level of each protein concentration (either secretory or intracellular) were measured (Figure 2D).

### 3.2. Single-Cell Analysis Using H1975 Lung-Cancer Cell Line

**Figure 3:**
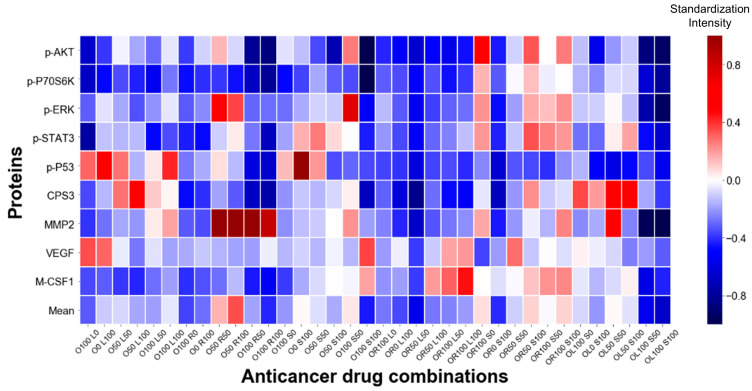
Heat map showing average protein concentration. The average protein concentration after anti-cancer drug treatment, for each drug, is illustrated using a heat map. O, Oximertinib; L, LY294002; R, Ruxolitinib; S, Selumetinib.

In this study, the changes in the levels of the nine aforementioned protein concentrations were identified, before and after treatment, in lung cancer cell lines. A total of three experiments were performed for each drug and a total of 172 cancer cells were used per experiment; the drug concentrations used in this study are detailed in Supplementary Materials Figures S2 and S3. The average protein concentration after drug treatment for each drug is illustrated using a heat map in Figure 3; in the map, blue indicates that the concentration decreases and red indicates its increases, with the value before drug treatment serving as the reference. The bottom row of the heat map shows the average of all the protein secretions, indicating that the concentration of proteins in OL100 S100 was the most downregulated (−0.67), and that in O50 R100 was the most upregulated (+0.34). A heat map showing the average protein secretion of the three experiments for all drugs is shown in Supplementary Materials Figure S6.

**Figure 4:**
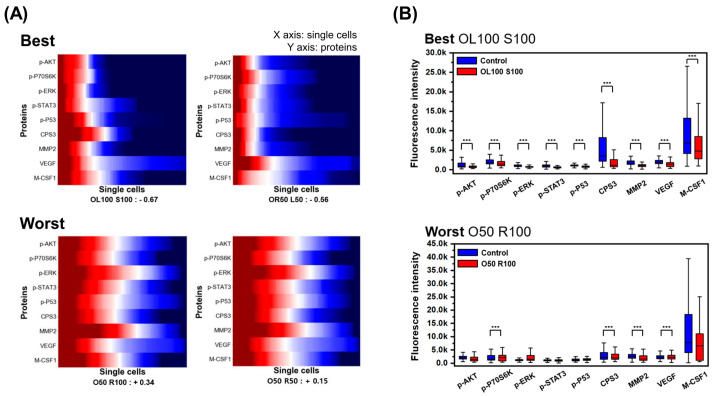
Single-cancer-cell proteomics analysis. (**A**) Heat maps showing protein concentration in best (OL100 S100, OR50 L50) and worst (O50 R100, O50 R50) drug treatment groups at the single-cell level are shown. (**B**) Box plots for protein concentration in best (OL100 S100) and worst (O50 R100) drug groups compared with control groups are shown. The number of asterisks corresponds to the following *p*-values. * *p*-value < 0.05, ** *p*-value < 0.01, *** *p*-value < 0.001. O, Oximertinib; L, LY294002; R, Ruxolitinib; S, Selumetinib.

Figure 4A shows the best drug groups as those with many blue areas (downregulation of target protein concentration) while the worst drug groups had more red areas (upregulation of target protein concentration) than the best groups at the single-cell level. Box plots are plotted in Figure 4B to compare the protein secretion of the drug and control groups. The best group (OL100 S100) showed significant decreases in all proteins compared to the control group while the worst group (O50 R100) showed inconsistent and fewer significant changes compared to the control group. To quantitatively identify changes in protein secretion, the mean protein changes in the two best and worst drug groups were evaluated (Supplementary Materials Figure S7). For the best drug groups, all proteins tended to decrease. However, the increases and decreases among proteins were inconsistent for the worst drug groups. In addition, histograms confirming the protein secretion distribution, in each single cell, for the best (OL100 S100) and worst (O50 R100) drug groups are shown in Supplementary Materials Figure S8; in these plots, the overall protein secretion distributions shift to the left (to lower levels) with treatment, for the best drug group, while those of the worst drug group remained largely unchanged.

**Figure 5:**
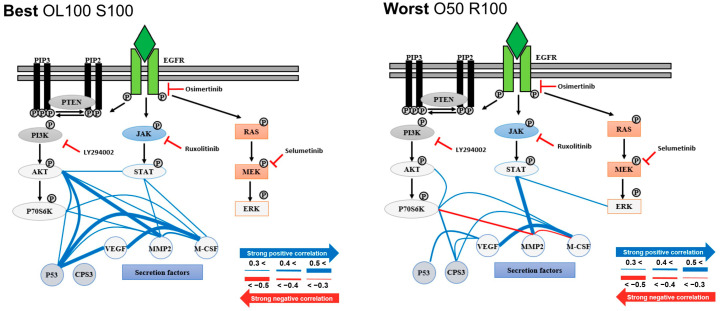
Correlation network for proteins in best (OL100 S100) and worst (O50 R100) drug groups. The blue line indicates a positive correlation between the two proteins and the red line indicates a negative correlation. The thickness of the line indicates the degree of correlation. O, Oximertinib; L, LY294002; R, Ruxolitinib; S, Selumetinib.

Furthermore, correlation analysis was performed to determine the correlation between the proteins in the best and worst drug groups (Figure 5). The best drug group had correlations of 0.3 or higher in 15 cases, while the worst drug group had the same result in 11 cases. Among the correlations, MMP2 and p-P70S6K, and MMP2 and M-CSF1 showed opposing results in the best and the worst drug groups (positive correlation in the best drug group and negative correlation in the worst drug group).

### 3.3. Evaluation of Heterogeneity

**Figure 6:**
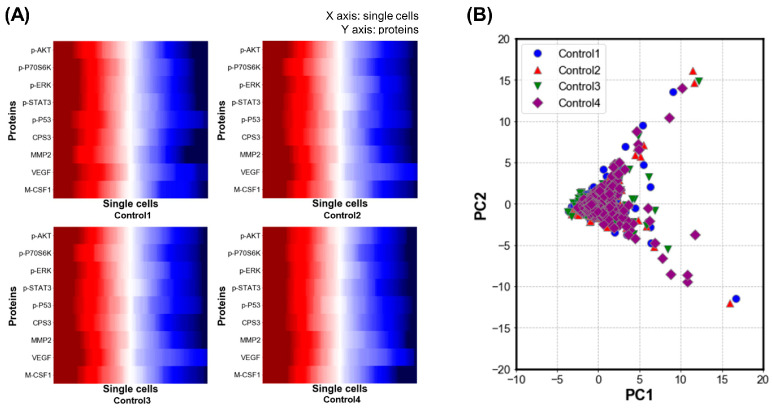
Cancer-cell heterogeneity evaluation. (**A**) Heat maps for protein concentration in four control groups at the single-cell level are shown. (**B**) Principal component analysis for the four control groups is shown.

To confirm the heterogeneity of cancer cells, before each anti-cancer drug treatment, the protein secretion data among the cancer cells were compared. Figure 6A shows the protein secretion of cancer cells in all four control groups (172 cancer cells per experiment, three experiments per control group). Figure 6B shows the PCA of protein secretion in the four control groups, which revealed slight differences between single cells in the four control groups.

**Figure 7:**
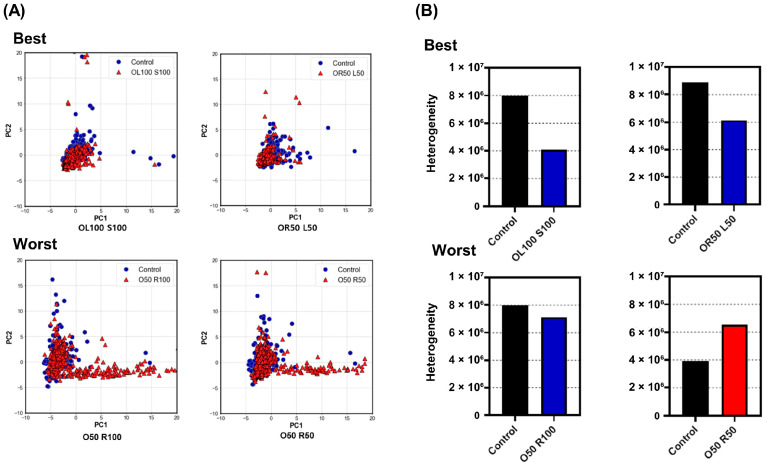
Cancer-cell heterogeneity evaluation. (**A**) Principal component analysis after best (OL100 S100, OR50 L50) and worst (O50 R100, O50 R50) drug group treatments, compared to the control group (before drug treatment). (**B**) Cancer cell heterogeneity after best (OL100 S100, OR50 L50) and worst (O50 R100, O50 R50) drug group treatments, compared to the control group (before drug treatment) via cluster analysis. O, Oximertinib; L, LY294002; R, Ruxolitinib; S, Selumetinib.

To identify changes in heterogeneity after anti-cancer drug treatment, PCA was performed on the best (OL100 S100, OR50 L50) and worst (O50 R100, O50 R50) drug groups, using the control groups as a reference (Figure 7A). After drug treatment, the distribution of single cells in the best drug groups was more condensed. In contrast, the distribution became more dispersed in the worst drug groups. Cluster analysis was performed to quantitatively evaluate the heterogeneity of cancer cells before and after anti-cancer drug treatment. Figure 7B shows that, compared to the control groups, the best drug groups (OL100 S100 and OR50 L50) reduced the heterogeneity by 48.99% and 30.76%, respectively, whereas the worst drug groups (O50 R100 and O50 R50) exhibited a 10.55% decrease and 66.79% increase in heterogeneity, respectively. Dendrograms from the cluster analysis of the control group and each drug group are shown in the Supplementary Materials Figure S9.

### 3.4. Comparison between Bulk and Single-Cell Assay

**Figure 8:**
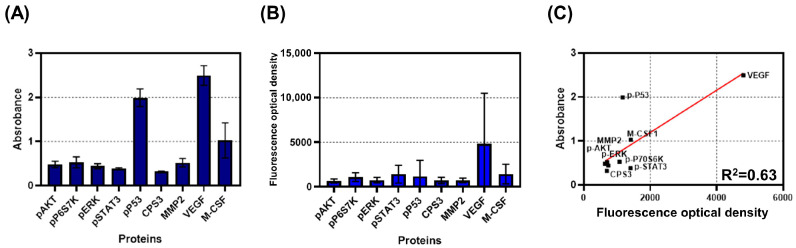
Comparison between bulk and singe-cell assays. (**A**) Protein concentrations from bulk assay are shown. (**B**) Protein concentrations from single-cell assay are shown. (**C**) Linear regression between bulk and single-cell assays is shown; the R^2^ value is calculated using this analysis.

Experiments were conducted to compare bulk and single-cell assays. The protein concentrations are shown in Figure 8A,B. In both cases, VEGF concentration was the highest. Linear fitting was performed for regression analysis of the concentrations from the two assays (Figure 8C); the obtained R^2^ value was 0.63.

## 4. Discussion

For cancer patients, it is important to overcome resistance to chemotherapy and to select appropriate dosages of anti-cancer drugs to reduce the side effects of chemotherapy [42]. This study was able to identify changes in protein secretion before and after drug treatment using the heat maps generated by single-cell proteomic analysis. For the worst drug groups, we observed an increase in p-ERK and MMP2 compared to the control groups (Figure 4B). Both proteins are known to be related to the progression and recurrence of lung cancer, with unfavorable outcomes [43,44]. Figure 3 demonstrates that our chip can evaluate the effects of anti-cancer drug combinations, as well as their dosages, providing a variety of information.

The heterogeneity of cancer cells can be divided into two categories. The presence of different cancer cell characteristics within the same tumor tissue is called intra-tumor heterogeneity while the same tumor tissue presenting with different characteristics in individual patients is called inter-tumor heterogeneity [6,45,46]. The device in this study was able to identify intra-tumor heterogeneity in lung cancer cells by evaluating the concentrations of nine secreted proteins. In addition, changes in heterogeneity before and after anti-cancer drug treatment were qualitatively and quantitatively verified through PCA and cluster analysis, respectively.

Obulkasim et al. compared the heterogeneity of cancer between a group of locally advanced esophageal adenocarcinoma patients who only underwent surgery and a group that underwent the same surgery as well as neoadjuvant chemotherapy. The group that underwent surgery and neoadjuvant chemotherapy exhibited decreased heterogeneity. The study outcomes suggested that a decrease in heterogeneity may contribute to a survival benefit [47]. In the present study, we found that the heterogeneity decreased when cells were treated with the most effective drugs, and the heterogeneity either decreased slightly or increased when the least effective drugs were used (Figure 7B). Inter-tumor heterogeneity could likely have been evaluated using this method if samples were obtained from patients.

Another notable aspect of cancer cell heterogeneity in this study is the difference in bulk and single-cell protein concentrations shown in Figure 8. The bulk and single-cell assay results showed some degree of similarity (R^2^ = 0.63) but were not entirely identical. This result demonstrates that, as mentioned in previous studies, bulk assays cannot fully represent population characteristics, resulting in some important information being overlooked [48,49,50]. Our device was able to provide heterogeneity information on single-cell proteomics, which could be obscured in bulk assays.

However, this study had some limitations. First, unlike the conventional method of identifying heterogeneity from a genomics perspective, this study confirmed heterogeneity from a proteomics perspective; however, it should be noted that protein expression cannot only be predicted by DNA and RNA expression [16,51]. In addition, since protein modification (methylation, phosphorylation, etc.) within the gene sequence is unknown, it is important to observe the correlation between proteins and cancer cell heterogeneity at the proteomic level [16,17,52]. Second, because this study requires a reasonably large number of cancer cells, it is difficult to obtain sufficiently large samples from liquid biopsies. Further, liquid biopsy sampling is an emerging method; however, it is not currently considered a standard protocol. Hence, invasive biopsies are necessary for an accurate diagnosis of heterogeneity [53,54]. Third, given that this study was performed using cell lines, its clinical usefulness has not been established. Further studies investigating the validity of the method, using animal experiments and samples from real clinical patients, may be required to demonstrate its feasibility in clinical applications.

## 5. Conclusions

Cancer changes constantly and becomes heterogeneous. Due to this heterogeneity, cancer varies from patient to patient and should be analyzed at the single-cell level for effective anti-cancer treatment. Based on microfluidic technology, our single-cell assay chip can manipulate multiple single cancer cells (1376 chambers), rapidly dissolve and wash cells using valves and pumps, and evaluate cellular responses to a combination of anti-cancer drugs by detecting secreted proteins and intracellular molecules using barcode immunoassays. This study was concerned with the qualitative and quantitative evaluation of the heterogeneity of cancer cells, by analyzing single-cell proteomics in lung cancer cell lines using our microfluidic single-cell assay chip. We proposed a new approach for the selection of anti-cancer drug combinations and doses, comparing changes in proteins and heterogeneity, before and after anti-cancer drug treatment. Subsequent preclinical and clinical trials should enable the development of this platform for patient-customized therapeutic applications.

## Data Availability

The data presented in this study are available in this article.

## Supplementary Materials

The following are available online at https://www.mdpi.com/article/10.3390/mi12101147/s1, Video S1: Driving method of the single-cell assay chip, Table S1: List of capture antibody identifications and product details used in this study, Figure S1: Schematic of immobilized antibody on a glass substrate, Figure S2: Relative cell proliferation rate with respect to drug concentration in lung cancer cell line, Figure S3: Result of MTT assay for different drug combinations, Figure S4: Schematic showing the architecture of the signal amplification, Figure S5: Mixing and washing efficiency, Figure S6: Heat map showing average protein concentration, Figure S7: Mean protein concentration change compared with control groups, Figure S8: Histograms showing protein secretion distribution, Figure S9. Cluster analysis dendrograms.
