# Supplementary material for: Microfluidic Single-Cell Proteomics Assay Chip: Lung Cancer Cell Line Case Study
Source: Micromachines (Basel). 2021 Sep 23;12(10):1147. doi: 10.3390/mi12101147 (PMC8541572; doi:10.3390/mi12101147)
Supplement: Supplementary file 1 [file micromachines-12-01147-s001.zip › micromachines-1368382-supplementary.pdf]

Microfluidic Single-Cell Proteomics Assay Chip: Lung-Cancer Cell Line Case Study

Yugyung Jung [1,¶], Minkook Son [1,¶], Yu Ri Nam [1,2], Jongchan Choi [3,4], James R. Heath [4], and Sung Yang [1,3*]

[1]Department of Biomedical Science and Engineering, Gwangju Institute of Science and Technolo gy (GIST), Gwangju 61005, Korea

[2]Department of Chemistry, Korea Advanced Institute of Science and Technology (KAIST), Daeje on 34141, Korea

[3]School of Mechanical Engineering, Gwangju Institute of Science and Technology (GIST), Gwangj u 61005, Korea

[4]Division of Chemistry and Chemical Engineering, California Institute of Technology, CA 91125, USA

¶These authors contributed equally to this work.

*Correspondence: syang@gist.ac.kr

Supplementary materials

Video S1. Driving method of the single-cell assay chip.

Table S1. List of capture antibody identifications and product details used in this study.

Figure S1. Schematic of immobilized antibody on a glass substrate.

Figure S2. Relative cell proliferation rate with respect to drug concentration in lung cancer cell line.

Figure S3. Result of MTT assay for different drug combinations.

Figure S4. Schematic showing the architecture of the signal amplification.

Figure S5. Mixing and washing efficiency.

Figure S6. Heat map showing average protein concentration.

Figure S7. Mean protein concentration change compared with control groups.

Figure S8. Histograms showing protein secretion distribution.

Figure S9. Cluster analysis dendrograms.

Table S1. List of capture antibody and anti-cancer drug product details used in this study.

**Identification**	Product details	Catalogue Number
**Capture antibody**		
**Reference**	ssDNA: 5′-NH2-C6-AAAAAAAAAAAAAGCCTCATTGAATCATGCCTA-3′<br>ss cDNA: 5′-Cy3-AAAAAAAAAAAAATAGGCATGATTCAATGAGGC-3′	Bioneer
**p-AKT`**	Hunan/Mouse Phospho-Akt1(S473), Duoset IC ELISA	DYC 2289C-2
**p-P70S6K**	Phospho-p70S6Kinase(T389) Duoset IC ELISA	DYC 896-2
**p-ERK1/ERK2**	Phsho-ERK1(T202/Y204)/ERK2(T185/Y187), Duoset IC ELISA	DYC 1018B-2
**p-STAT3**	Human/Mouse Phospho-STAT3 (Y705), Duoset IC ELISA	DYC 4607B-2
**p-P53**	Human Phospho-p53(S15), Duoset IC ELISA	DYC 1839-2
**Cleaved Caspase-3**	Human/Mouse Cleaved Caspase-3(Asp175), Duoset IC ELISA	DYC835-2
**MMP2**	Human MMP2, Duoset ELISA	DY 902
**VEGF**	Human VEGF, Duoset ELISA	DY 293B
**M-CSF1**	Human M-CSF, Duoset ELISA	DY 216
**Anti-cancer drug**		
**Osimertinib (AZD9291)**	Osimertinib (AZD9291) is the mutant-selective EGFR inhibitor.	S7297
**LY294002**	LY294002 (SF 1101, NSC 697286) is the first synthetic molecule to inhibit PI3Kα/δ/β.	S1105
**Selumetinib (AZD6244)**	Selumetinib (AZD6244, ARRY-142886) is the potent, highly selective MEK inhibitor.	S1008
**Ruxolitinib (INCB018424)**	Ruxolitinib (INCB018424) is the first potent, selective, JAK1/2 inhibitor.	S1378

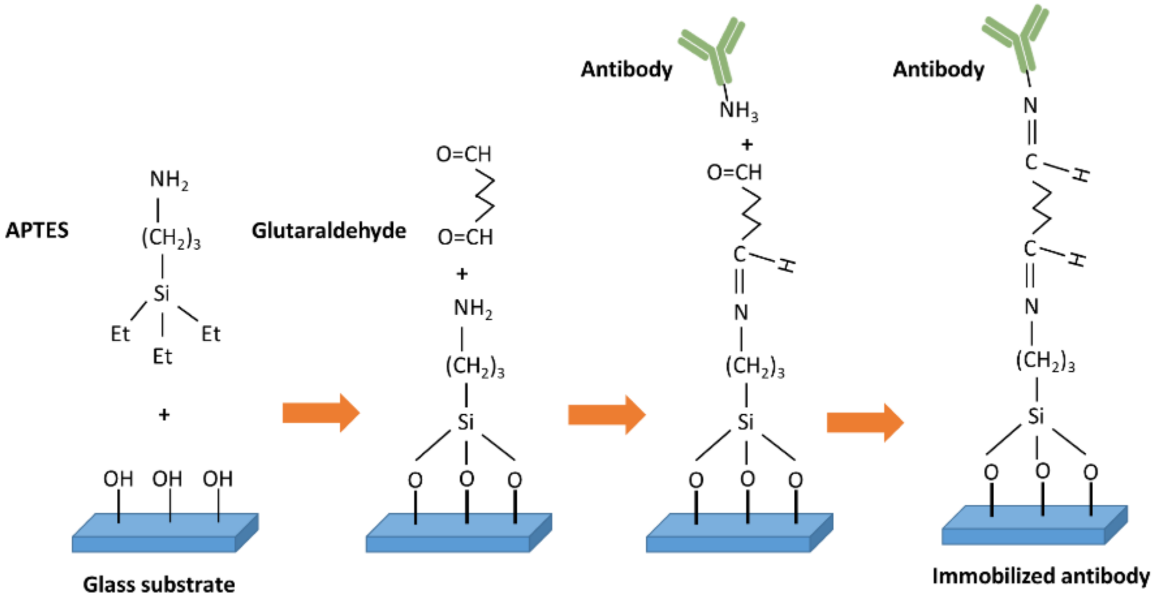

Figure S1. Schematic of immobilized antibody on a glass substrate. To immobilize the capture antibodies on a glass substrate, its surface properties must be modified. First, the glass substrate was treated with oxygen plasma (100 W, 20 sccm, 30 s). Then, by treating the substrate with 3-aminopropyltriethoxysilane (APTES) solution (3% v/v) in ethanol overnight, the surface was converted to an amine surface; by using glutaraldehyde (10% v/v) solution, the amine surface can be turned into an aldehyde one. The amine groups of the capture antibody will bind with the aldehyde groups.

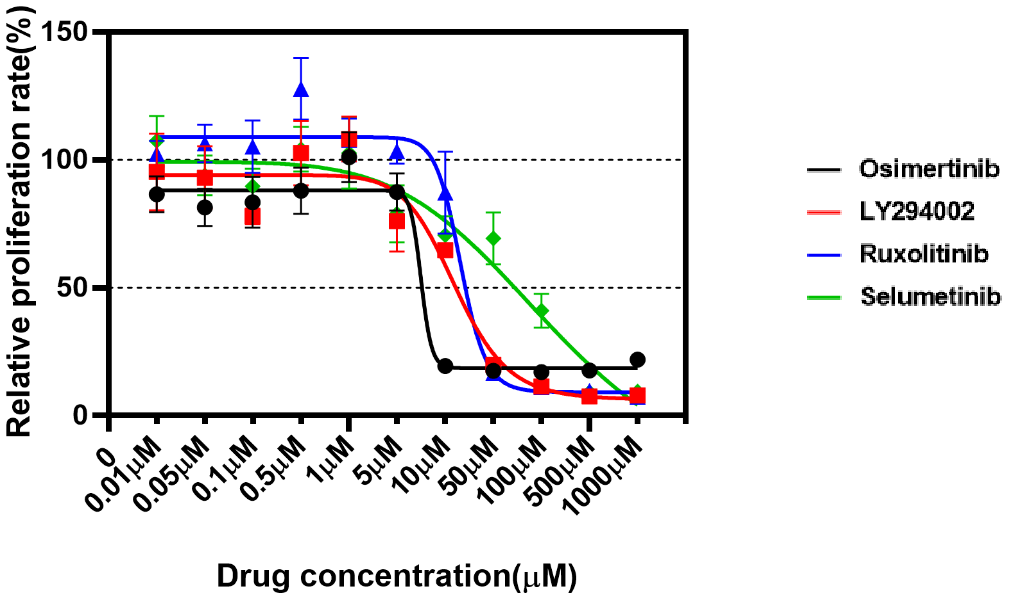

Figure S2. Relative cell proliferation rate with respect to drug concentration in lung cancer cell line. In this study, a general protocol for an MTT assay was used to evaluate the half-maximal inhibitory concentration (IC_{50}) of each drug (O, Oximertinib; L, LY294002; R, Ruxolitinib; S, Selumetinib) and drug combination for the H1975 lung-cancer cell line.

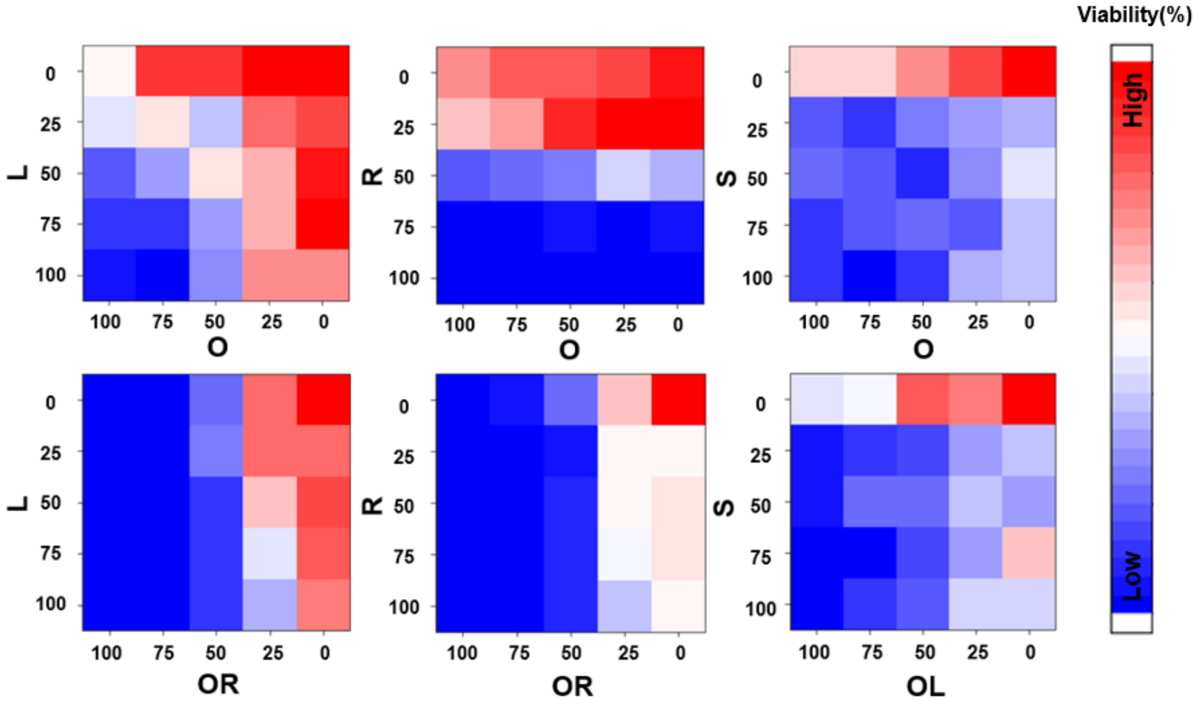

Figure S3. Result of MTT assay for different drug combinations. Relative cell proliferation rate is visualized by a heat map. Red represents high viability while blue does low viability. For the dual-drug combinations, the IC_{50} concentration of each single drug was set at 100% and diluted with culture medium (RPMI-1640) to produce 0%, 25%, 50%, and 75% relative proliferation values. O, Oximertinib; L, LY294002; R, Ruxolitinib; S, Selumetinib.

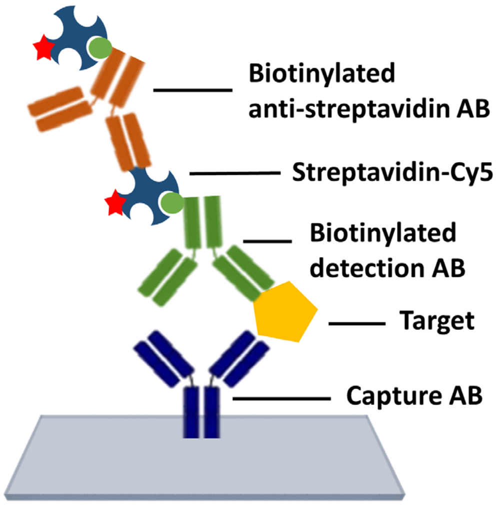

Figure S4. Schematic showing the architecture of the signal amplification. Biotinylated anti-streptavidin antibody (AB) followed by a streptavidin-cyanine5 (Cy5) was used to increase the fluorescence signal.

The mixing efficiency(M) can be calculated by the formula as follows,

$$M = 100 \times (1 - \sqrt{\frac{1}{n}\sum_{i=1}^{n}(\frac{k_i - \bar{k}}{k})^2}) \qquad (1)$$

Where M stands for the mixing efficiency, n is the total number of sampling points, ki is the mole fraction distribution over the whole cross-section, and $\bar{k}$ is the average mole fraction. Then, mixing efficiency ranges from 0 (0% mixing) to 1 (100% mixing) by the formula.
The washing efficiency can be calculated by the equation as shown in eq. (2)

$$\text{Washing efficiency} = \frac{(Initial\ intencity - Sampling\ intensity)}{Initial\ intensity} \times 100 \qquad (2)$$

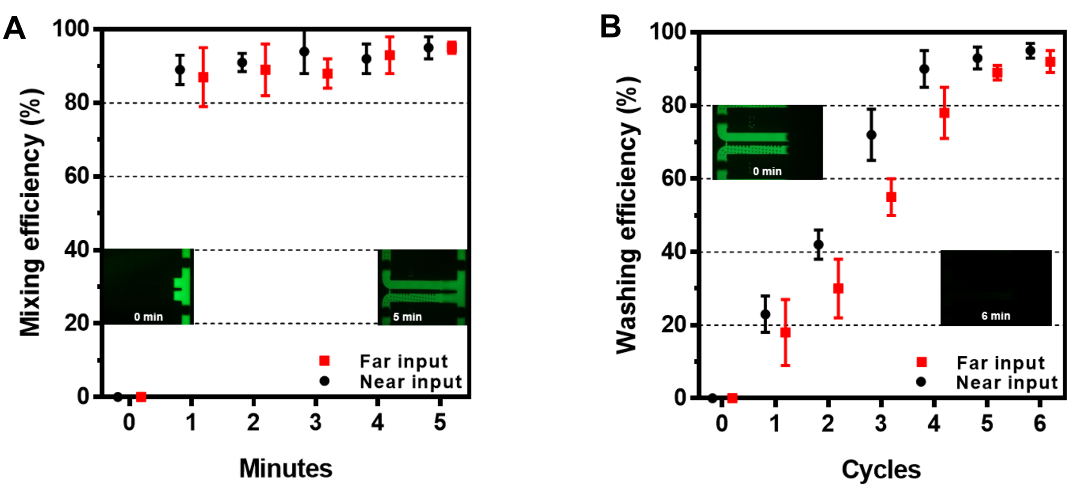

Figure S5. Mixing and washing efficiency. The rapid single-cell lysis and individual chamber washing were achieved not only in a single chamber but also in entire assay chambers, without any fluidic interference or contamination among the chambers (A) Mixing efficiency with respect to time is shown. (B) Washing efficiency with respect to washing cycles is shown.

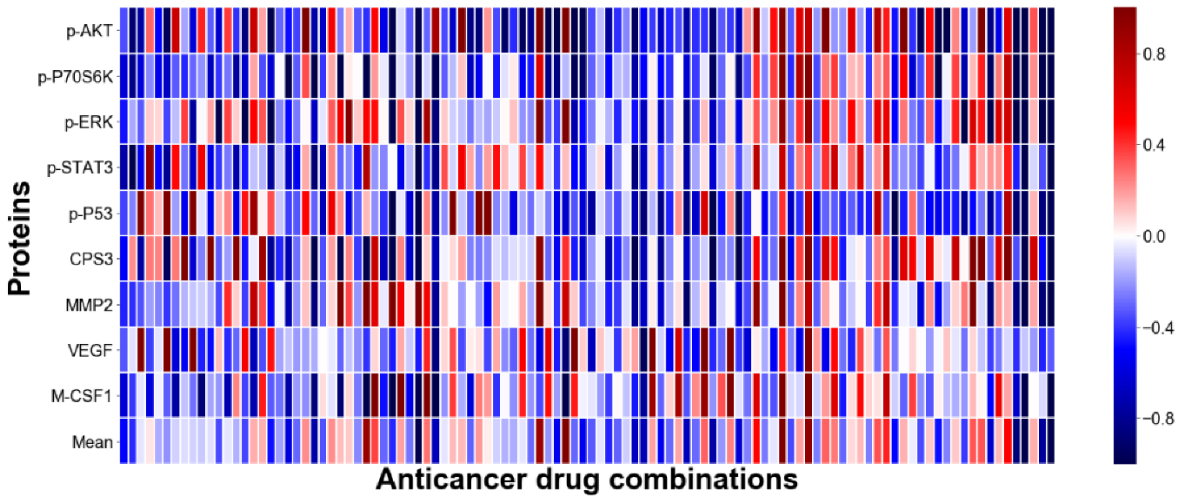

Figure S6. Heat map showing average protein concentration. The average protein concentration after anti-cancer drug treatment of three experiments, for all the drugs, is illustrated using a heat map.

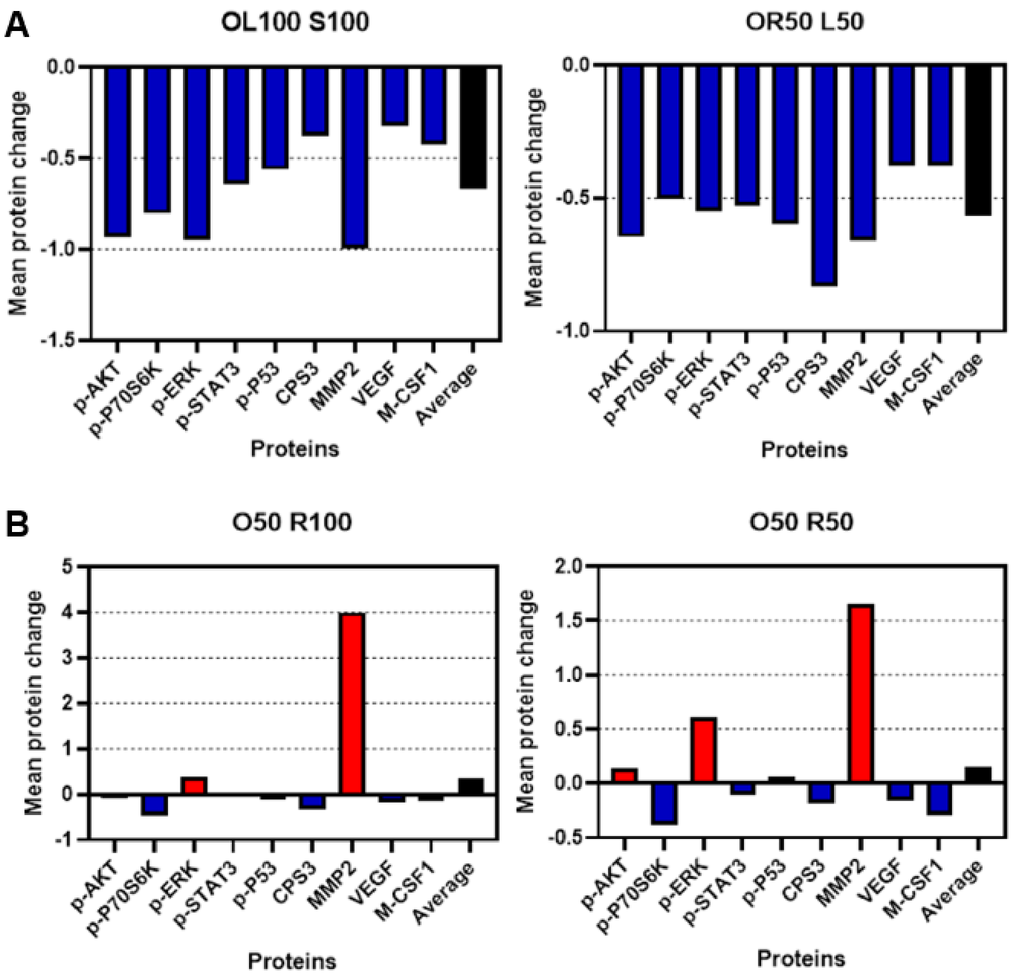

Figure S7. Mean protein concentration change compared with control groups. To quantitatively identify changes in protein secretion, the mean protein changes in the two best and worst drug groups were evaluated. (A) Best drug groups (OL100 S100, OR50 L50). (B) Worst drug groups (O50 R100, O50 R50). O, Oximertinib; L, LY294002; R, Ruxolitinib; S, Selumetinib.

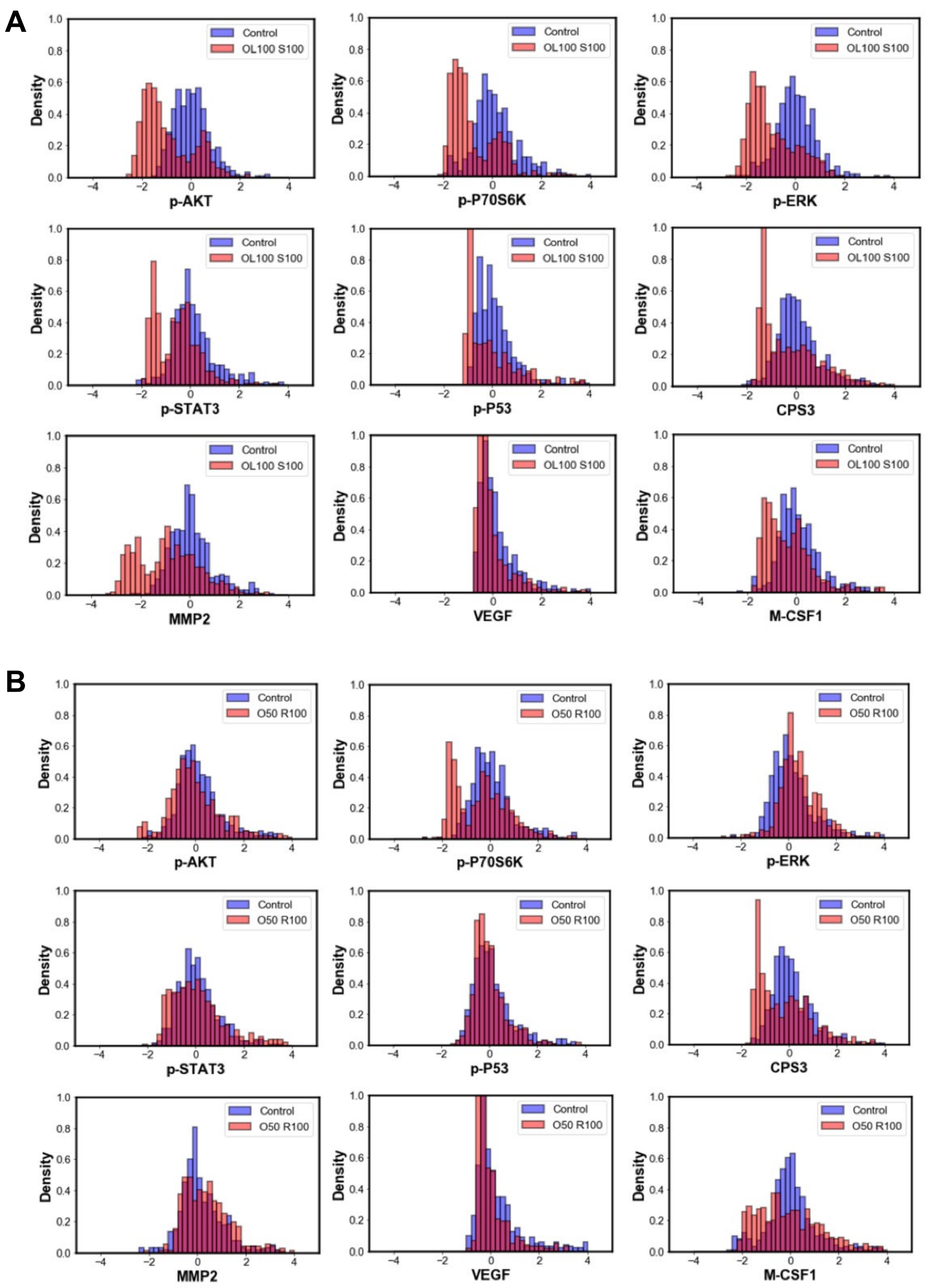

Figure S8. Histograms showing protein secretion distribution. The histograms confirming the protein secretion distribution at the single cell level for the best (OL100 S100) and worst (O50 R100) drug groups are shown. (A) Best drug group (OL100 S100). (B) Worst drug group (O50 R100). O, Oximertinib; L, LY294002; R, Ruxolitinib; S, Selumetinib.

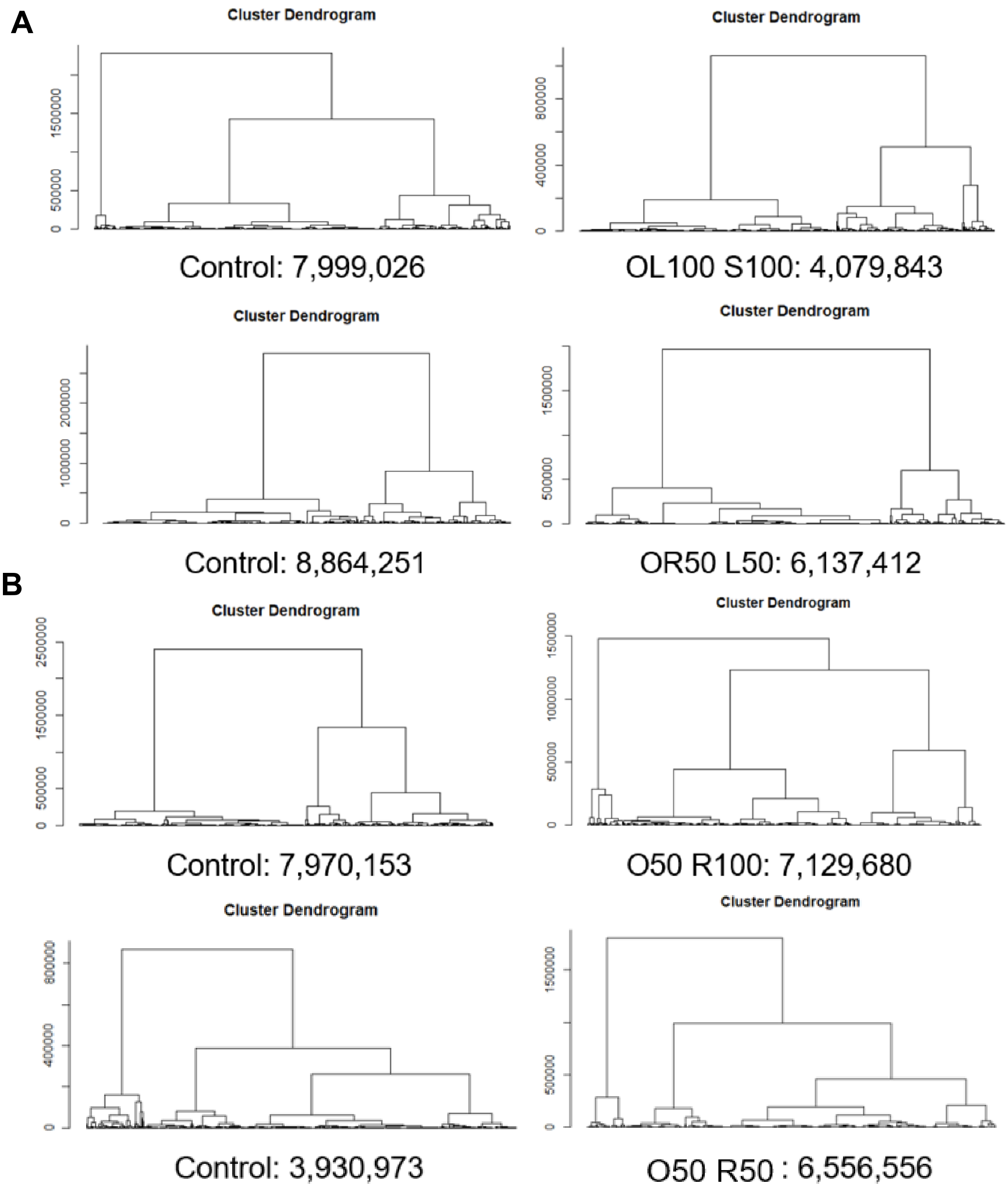

Figure S9. Cluster analysis dendrograms. The dendrograms are shown for best and worst drug groups compared to the control group. (A) Best drug groups (OL100 S100, OR50 L50). (B) Worst drug groups (O50 R100, O50 R50). O, Oximertinib; L, LY294002; R, Ruxolitinib; S, Selumetinib.
